# Sabbath Days

**Published:** 1869-08

**Authors:** 


### SABBATH DAYS.

Many a man has confessed under the gallows, that his downward progress began with misspent Sabbaths. Upon investigation, it will be often found, that the first steps taken were in what many call "innocent recreations," taking a drive, wandering in fields, loiterings by the river side, or visiting neighbors.

At home, or at church, are the places for spending the hours of the sacred day; especially is it the way of safety for young people—safety from the grog-shop, the engine-house, and the chambers of her whose ways go down to death: and how much of bodily disease are traceable directly to these three places, to say nothing of moral corruptions, any city physician, of even moderate practice, has daily cognizance.

One of the ways of saving persons from these calamities is, to offer facilities for spending at least some of the hours of the Sabbath in religious worship. To make this practical in a single point of view, we state an experience of our own within a month: The afternoon service by our own minister being necessarily omitted, we went to the next church in the same street at the usual hour of half past three o'clock. The bell was ringing, the church doors were open, but beyond one old lady taking her seat, there was not a living creature to be seen. The bell tolled on. In the course of a quarter of an hour single individuals began to drop in. We made inquiries of several as to the hour of service; we had asked near a dozen persons, not one of whom knew anything. The questions came pouring in upon us: Who preaches here? Is there any service this afternoon? What time does church call? Is this an Episcopal Church? Soon there was a crowd of well-dressed men and women standing about the door, looking in, and looking around. At last, persons began to come, who, by their direct passing on, seemed to be at home; but neither volunteering information or a seat to any of the standing company; the crowd increased, and the bell tolled on. As near half an hour is a long time for a professional man in a large city to stand with his finger in his mouth, we concluded we would pass on to some more hospitable vestibule, and visited two churches in succession, both of which were closed, to wit, on the third day of May.

The derelict church occupied a frontage of some two hundred feet on Fifth Avenue.

The practical thought occurred to us as the people came and went away, that for the sum of two dollars, a neat frame might have been placed against the wall in the vestibule, stating, first, the hours of service, and then designating some portion of the building where strangers might seat themselves, without waiting for the sexton to get through his half hour's bell-tolling.

We might further suggest, that a "lot" be sold off, the interest on the proceeds of which should be appropriated annually to the hire of two active young men, whose business it should be, while the sexton was tugging at the bell-rope, to answer the inquiries of strangers, and courteously show seats to such as wanted them. We may also add, and that too from our own experience as a world's traveller, our conviction of the great convenience it would be to travellers to see in the hotels a kind of church directory, in a neat frame, near the clerk's desk or registry-book, stating where, and at what hours and days, religious worship was held. Each of the half dozen principal denominations could have a separate column, all in a frame of a single square foot. We once walked the streets of London for a full hour on a beautiful Sunday morning in search of a Methodist or Presbyterian congregation, asked every police-officer we saw, without accomplishing our object.

We know very well, that in any of our large cities, there is not a night or a Sabbath day which does not find many strangers who would very gladly avail themselves of the opportunity of listening to some favorite or celebrated clergyman, if they knew where and when to go. Inexperienced persons may say: "It is easy to ask the clerk." That might answer in a small town: but in a city, it would be a hopeless work. In our large hotels, especially in Broadway, there is only one question that is either definitely, courteously, or correctly answered; and that is, the amount of your bill. New York physicians know, by experience, the difficulty of finding a person who has sent for them professionally. The flash hotel of Broadway, where people from the country will "put up" at, for the sake of "having it said" they stopped there, is notorious for this inattention. We have no idea that there is any malice in it, but simply indifference; they know nobody, except by the number of the room occupied:

General SCOTT is number "twenty," and TOM THUMB is number "twenty-two," and he is most regarded who has the most extras. These things ought not to be, but they are; and we adduce them as illustrations of the policy of having a church directory in an ornamental frame, hung up in each of the prominent hotels of our cities. We believe its practical effect would be to save many persons, in the course of a year, from falling into temptation and a snare, and disreputable disease.

